# Rare disease landscape in Brazil: report of a successful experience in inborn errors of metabolism

**DOI:** 10.1186/s13023-016-0458-3

**Published:** 2016-06-10

**Authors:** Roberto Giugliani, Filippo P. Vairo, Mariluce Riegel, Carolina F. M. de Souza, Ida V. D. Schwartz, Sérgio D. J. Pena

**Affiliations:** Medical Genetics Service, Hospital de Clínicas de Porto Alegre, Rua Ramiro Barcelos, 2350, 90035-903 Porto Alegre, RS Brazil; Department of Genetics, UFRGS, Porto Alegre, RS Brazil; Postgraduate Program in Genetics and Molecular Biology, UFRGS, Porto Alegre, RS Brazil; Laboratório de Genômica Clínica da Faculdade de Medicina and Departamento de Bioquímica e Imunologia, Universidade Federal de Minas Gerais, Belo Horizonte, MG Brazil

**Keywords:** Brazil, Genetic Diseases, Inborn Errors of Metabolism, Rare Diseases

## Abstract

Brazil is a country of continental dimensions, with many social inequalities. The latter are reflected on its health system, which comprises a large public component called SUS, a small paid health insurance component and a third very small private component, in which patients pay personally for medical services. Seventy five percent of the population depends on SUS, which thus far does not provide adequate coverage for genetic medical procedures. In 2014, SUS introduced the “Policy for the Integral Attention to Subjects with Rare Diseases”, establishing guidelines for offering diagnosis and treatment. The policy defines the two main axes, genetic and non-genetic rare diseases. In this fashion, public genetic services in SUS will be installed and funded not by themselves, but as part of the more general policy of rare diseases. Unfortunately, up to now this policy is still depending on financial allowances to be effectively launched. In this article, our intention was to describe activities developed in the area of inborn errors of metabolism by a Brazilian reference center. In spite of the lack of support of SUS, thousands of Brazilian families affected by rare genetic metabolic disorders, and many health professionals from all regions of Brazil, already have benefited from the services, training programs and research projects provided by this comprehensive center.

## Background

### Brazilian health system

Brazil is a federative republic with an area of 8.5 million km^2^, covering 47 % of South America. With an estimated population of more than 200 million individuals in 2015, Brazil is the world’s fifth most populous country. Unfortunately, its colossal size and large population are matched by huge regional and social inequalities.

These inequalities are reflected in the Brazilian health system, which, as described by Paim et al. [12], is composed of three subsectors: the public subsector (SUS), in which services are financed and provided by the state at the federal, state, and municipal levels, the private (for-profit and non-profit) subsector, in which services are financed in various ways with public or private funds, and the private health insurance subsector, with different forms of health plans, varying insurance premiums, and tax subsidies. The private health plan and insurance market is concentrated in the southeast region, where 61.5 % of health companies are based. According to Paim et al. [12], 18.4 % of the population has private insurance and 6.1 % have insurance for civil servants.

When we were asked to write about rare disease medical research and services in Brazil, we were at a crossroads. If we opted for describing all components of the rare disease health system in the different cities of the country we might end up, because of size limitations of this article, with a confused low resolution picture, *à vol d’oiseaux*. Rather than doing this, we decided to focus on research and services offered by a reference center in inborn errors of metabolism. We would like to make clear that many other similar high-quality research and service centers working on rare diseases, including on inborn errors of metabolism, exist around the country. However, we had no intention in being comprehensive, but instead chose to exemplify current practice focusing our article on a successful and quite unique experience in the rare disease field.

### Rare disease policies of the public Brazilian health system (SUS)

As previously mentioned, roughly 25 % of the Brazilian population has some type of health insurance coverage. The private health insurance companies are regulated by the National Supplementary Health Agency (ANS), a federal government organization established in 2000. Guidelines for coverage of several laboratory procedures for genetic diagnosis (genomic cytogenetics, biochemical and molecular techniques, particularly sequencing, and prenatal diagnostic tests) by the Insurance Companies were issued in January 2014 [14] and although this was a significant improvement in care, coverage is still limited in several areas.

Yet, the vast majority of the population depends exclusively on the public health system (SUS), which was created by the Brazilian Federal Constitution in 1988 and is organized by the Ministry of Health, with subsystems in each Brazilian State and city. SUS is one of the largest public health systems in the world, includes most aspects of health care, from outpatient care to organ transplantation, and proposes guidelines to ensure full, universal and free-of-charge medical access for the entire Brazilian population. In addition to offering appointments, medical exams and hospitalizations, SUS also promotes immunization campaigns, prevention and health monitoring, such as food and medication control and medicament registration. SUS so far does not provide adequate coverage of genetic medical procedures, with the exception of newborn screening, standard karyotype and very few other tests.

As described by Passos-Bueno et al. [14], in 2014 SUS introduced the “Policy for the Integral Attention to Subjects with Rare Diseases” in Brazil, establishing guidelines for offering comprehensive care (diagnosis, treatment and/or long term management) to individuals affected by rare diseases in the public unified health system (the same definition of World Health Organization for rare diseases, as those affecting less than 65 out of 100,000 individuals, was used). This policy defines an annual plan of action and financial and logistical support, and envisages the establishment of a national database (important for facilitating the access to high cost drugs, genetic tests, for instance) and the creation of reference treatment centers. These centers should be able to evaluate patients, perform genetic testing procedures, diagnose, treat and offer genetic counseling [15].

The policy defines the two main axes, genetic and non-genetic rare diseases. In addition, it divides the genetic rare diseases in 3 groups: congenital anomalies & late-onset diseases, intellectual disability and metabolic disorders. In this fashion, genetic services in SUS will be installed and funded not by themselves, but as part of the more general policy of rare diseases.

The Ministry of Health has promised to include in the care of 15 million Brazilian patients afflicted with eight thousand rare diseases the attendance by specialists in reference centers, including internet, telephone and video conference consultations [16]. Thus, at least in paperwork, Brazil has designed a potentially appropriate policy for dealing with rare diseases, which will finally bring medical genetics to the public health system. Unfortunately, up to now, the policy has not left the printed page and it is still depending on financial adjustments to be effectively launched. However, considering the many budget cuts being enforced to deal with the difficult state of the Brazilian economy in the mid of the 2010 decade, the effective implementation of this policy may still take a while.

## Activities of a reference center in inborn errors of metabolism

Hospital de Clínicas de Porto Alegre, located in the capital city (Porto Alegre) of the Southernmost state of Brazil (Rio Grande do Sul), has the Medical Genetics Service (MGS-HCPA) which is a reference center for rare diseases well known in the continent, being recognized since 2004 as WHO Collaborating Center for the Development of Medical Genetic Services in Latin America (WHO-CC). Among its many activities, we will focus in the services, research and education in the area of inborn errors of metabolism provided by MGS-HCPA.

### Brazilian networks for the investigation of inborn errors of metabolism

The training of specialists in medical genetics in Brazil, which started in the 1970’s and has been growing steadily since then, the inclusion of genetics in the medical practice of several specialties around the country and the fact that the technologies for genetic analysis became more widely available and accessible, created a demand for information on diagnosis and management of genetic conditions.

Nevertheless, medical specialists on rare conditions, such as inborn errors of metabolism, are only available in few centers around the country, usually public university hospitals. As mentioned before, the Brazilian public health system (SUS) does not reimburse diagnostic tests for most genetic diseases, particularly for the inherited metabolic conditions. Being a comprehensive genetic service with multiple laboratories located inside a top rank general university hospital and, due its particular expertise in inborn errors of metabolism, the MGS-HCPA was receiving in the beginning of this century a huge number of such requests for investigation of pediatric and adult patients, coming from public and private medical services from all Brazilian regions.

Prenatal diagnosis is restricted in Brazil, as pregnancy interruption due to fetal disease is not legally allowed, except cases of anencephaly. Regarding newborn screening (NBS), despite the Ministry of Health sponsoring a program available for each Brazilian baby at no charge, it is limited to only 6 conditions (phenylketonuria, congenital hypothyroidism, hemoglobin disorders, cystic fibrosis, biotinidase deficiency and congenital adrenal hyperplasia), and unfortunately most of the IEMs end up escaping newborn detection (more comprehensive NBS programs are available to the 25 % of population which subscribes private insurance policies).

Based in the successful experience of SIAT (Teratogen Information System) set up at MGS-HCPA in the 1990’s, in 2001 we decided to organize the way we deal with these requests and set up the Information Service on Metabolic Diseases (SIEM). When we realized that an information service on IEM could not move forward without diagnostic facilities available, we subsequently created the MPS Brazil Network, the IEM Brazil Network, the NPC Brazil Network, and the LSD Brazil Network.

In the next sections, we will briefly describe these initiatives, which are maintained with public grants and private donations, including unrestricted grants from companies.

Each of these networks has a Coordinating Center (headquarters, which hosts the information hub and the main laboratory facility), Associated Centers (services that have capability to provide comprehensive clinical services and/or perform a limited set of tests) and Participant Centers (any medical service that needs testing and/or information). Their headquarters is located at the MGS-HCPA, where is based their executive group (General Coordinator, Technical Staff, Operational Manager, Administrative Manager). Important decisions are shared with the Associated Centers, and a general meeting is held at least once a year during a congress or symposium.

#### The information service on metabolic diseases (SIEM)

The SIEM was created in 2001, initially as a toll-free telephone service available to any health professional in Brazil who was in need of information about how to obtain the diagnosis in a case with suspected metabolic disease (typical questions: which tests to request? which samples to collect? to which lab should the samples be shipped? what specimens to obtain immediately post-mortem?). In addition, information about emergency management of suspected cases and a list of specialized professionals in the geographic area could be provided upon request. The telephone is available 24/7, with a member of the technical staff taking the calls from Monday to Friday in business hours, otherwise an answering machine records the message and a follow-up call is made in no more than 24–48 h. SIEM progressively started to work more and more with electronic requests that could be filled at the website (www.siem.ufrgs.br, Portuguese and Spanish versions available) [4]. Until the end of 2015, SIEM received 2,720 requests for diagnostic help (not including requests for information and follow-up). Of these request, 1,763 required further investigation, and a diagnosis of an inborn error of metabolism being eventually confirmed in 264 cases (15 %).

#### The inborn errors of metabolism (IEM) Brazil network

**Fig. 1:**
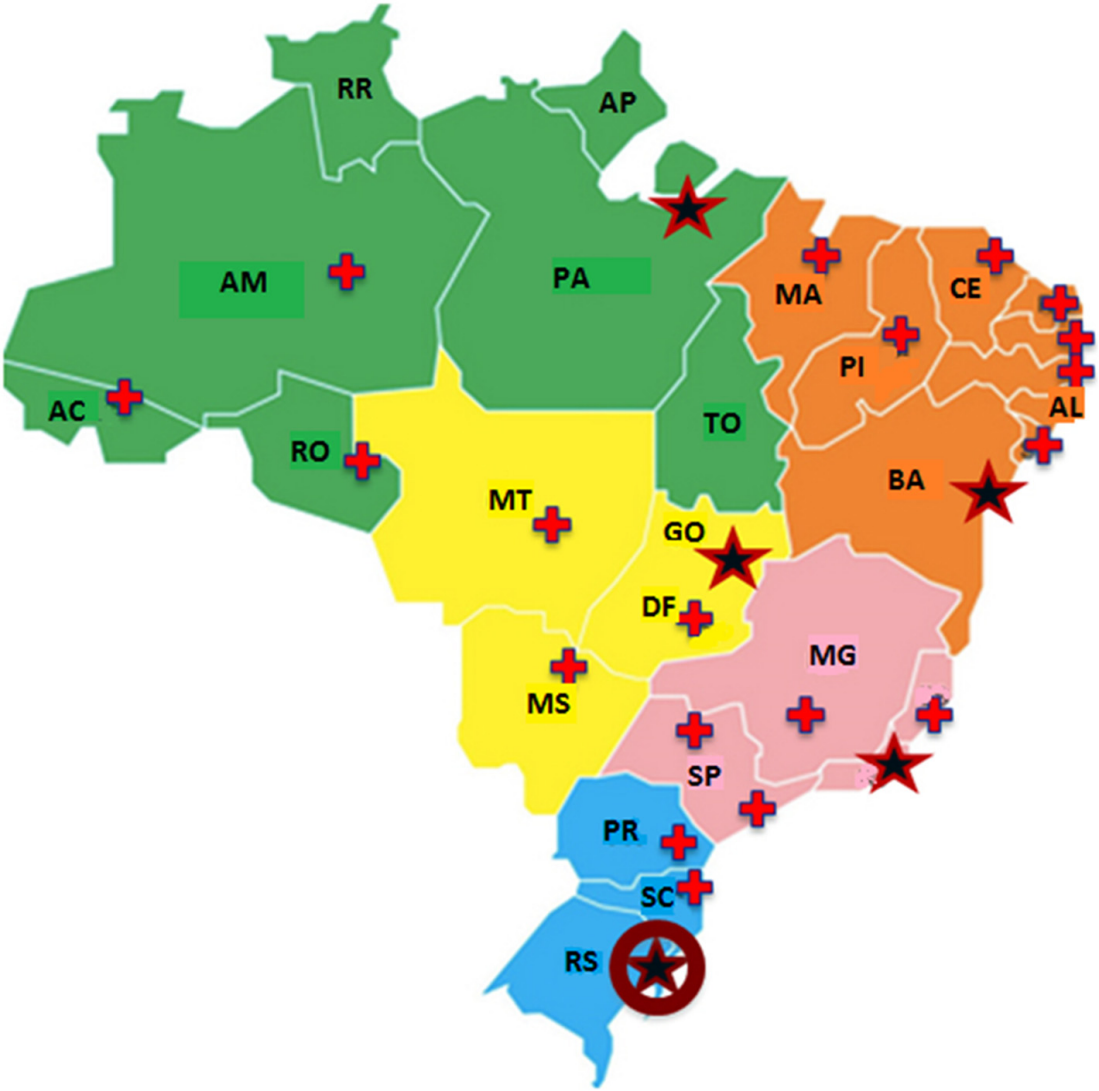
Distribution of centers of the MPS Brazil Network; crosses indicate “Participant Centers”; stars indicate “Associated Centers” (at least one in each Brazilian region, which have different colors) and the star with a circle indicate the “Coordinating Center” location

The IEM Brazil Network (www.eim.ufrgs.br) was created as a diagnostic arm of SIEM, based on a very well established diagnostic laboratory (Wajner et al. [18]). Every service dealing with patients with confirmed or suspected metabolic diseases could become affiliated to the IEM Brazil Network, if the patient is assisted within the public health service (SUS). This service allowed medical genetics, pediatrics, child neurology, among other clinics, from all over the country, to investigate suspected patients in a comprehensive way, with sophisticated analysis of amino acids, organic acids, acylcarnitines, very long chain fatty acids, and many other metabolites. Until 2015, the IEM Brazil Network performed 73,440 tests in 19,982 patients from all 27 Brazilian states, having diagnosed 698 cases of inborn errors of metabolism. The most frequent groups of conditions diagnosed were organic acidemias (19 %), peroxisomal disorders (17 %), amino acid disorders (16 %), energy metabolism disorders (13 %), carbohydrate metabolism disorders (8 %) and urea cycle defects (6 %). These diagnoses include many treatable conditions, emphasizing the importance of the SIEM-IEM Brazil Network combined strategy. Figure 1 shows the geographical distribution of the IEM Brazil network members.

#### The LSD Brazil network

A particular group of genetic metabolic conditions—the lysosomal storage diseases, LSDs—has received a disproportionate attention among the IEM, compared to their relative size (there are around 60 LSDs among the 600 IEM), as specific treatments—as enzyme replacement therapies, substrate reduction therapy and molecular chaperones—became available for many of these conditions. As the investigation for LSDs usually follows a relatively different strategy compared to the investigation for the other IEM, we decided to create a separate network: the LSD Brazil Network (www.dld.ufrgs.br). This network has investigated 30,576 patients until 2015, in whom 85,613 tests were performed. The number of cases diagnosed in the cases referred was 1,329 (not including mucopolysaccharidoses and Niemann-Pick type C disease, handled by other networks as described in the following topics). The most frequent conditions (over than 50 cases each) were metachromatic leukodystrophy (241 cases), Gaucher disease (197 cases), Niemann-Pick A/B diseases (97 cases), Fabry disease (83, considering only males) and Tay-Sachs disease (68 cases). This network has been also involved in screening programs in high-risk patients [11, 13].

#### The MPS Brazil network

The headquarters of the networks described in this section is well recognized by its work in lysosomal storage diseases and, particularly, in the mucopolysaccharidoses (MPS), started well before ERT became available. The huge number of requests for biochemical and genetic testing of cases with suspected MPS led to the creation of a separate network for this group of diseases, the MPS Brazil Network (www.mps.ufrgs.br), which is now well known worldwide for its diagnostic, research and education contributions.

The MPS Brazil Network has associated centers in all Brazilian regions and “participant centers” in all Brazilian states. It receives around 65 requests per month for investigation of MPS, with an average of 6 new cases of MPS diagnosed each month. The MPS Brazil network provides a comprehensive investigation for all MPS types, and all diagnosed cases are fully characterized in terms of biochemical phenotype and genetic mutations. In addition to the diagnostic service provided, the MPS Brazil Network has a toll-free line to provide information for families and health professionals, publishes a quarterly newsletter, promotes educational meetings and has actions to increase awareness in the community about MPS diseases [9]. The data obtained in the first 1,000 patients diagnosed (30 % MPS II, 23 % MPS VI, 20 % MPS I, 11 % MPS IV A, 7 % MPS III B and 9 % distributed among the other MPS types) was instrumental for the better understanding of the MPS epidemiology in Brazil and for the planning of preventive and management measures.

#### The NPC Brazil network

As Niemann-Pick type C (NPC) disease requires for its diagnosis an invasive technical tool, the Filipin staining test performed in fibroblasts, we set up an independent network to deal with the protocol to diagnose this challenging disease. The NPC Brazil Network (www.ufrgs.br/geneticahcpa/npc/) provides a comprehensive laboratory approach, including screening tests (chitotriosidase, oxysterols), diagnostic tests (Filipin), differential diagnosis (enzyme assays for Gaucher, Niemann-Pick A/B, acid lipase deficiency, and others) and confirmatory genetic analysis of *NPC1* and *NPC2* genes [10]. This network identified, until the end of 2015, 75 cases of NPC disease in Brazil, coming from all five Brazilian regions, the vast majority (96 %) being caused by mutations in the *NPC1* gene, as expected.

#### Research projects

The intense activity developed by the diagnostic networks on metabolic diseases made available a large number of samples and associated patient information. Basic epidemiologic data became available to allow the identification of prevalent regional targets for investigation. High numbers of patients for specific rare diseases enabled centers associated to these networks to develop basic and applied research projects and to participate in international multicenter clinical research protocols, in addition to developing original clinical research projects (a clinical trial to develop a innovative therapy approach for MPS I, developed in-house, was recently approved and funded by the Brazilian Ministry of Health).

Applied research has been also performed with the aim of improving laboratory diagnosis, especially to develop diagnostic protocols more suitable for developing countries, where conditions for collection and transportation of samples are far from ideal. The innovative method to assay lysosomal enzymes in leucocytes impregnated in filter paper is one example of this activity [5].

Several projects aiming to better understand the basic pathogenic mechanisms of several genetic metabolic diseases are being developed, boosted by the relatively high number of patients and samples considering that the conditions are rare diseases. The projects involving the study of oxidative stress pathways are one example of this type of research activity [3] as is the project involving several Brazilian services, coordinated from Porto Alegre, aiming to better understand the expression of Fabry disease in females. Another example of the experimental research is the one that uses in-house technology developed with modification of mammalian cells to overexpress specific enzymes, being the cells trapped into alginate capsules, which are implanted in animal models to treat specific conditions (MPS I has been the main target, so far) [1]. Currently, innovative techniques of genome editing as the CRISPR-Cas9 system are being established to treat MPS I as well.

The diagnostic activity of the networks has allowed the identification of clusters of genetic metabolic diseases in specific areas, which became targets for population-based studies, developed in partnership with the Brazilian Institute of Population Medical Genetics (INAGEMP) [6].

Finally, this activity has enabled Brazil to participate in several international multicenter clinical research protocols, related to the natural history or therapy development in rare genetic diseases This participation allowed many Brazilian groups to develop skills and facilities for clinical research, which eventually enabled some centers to develop original clinical research projects and request funding (already granted in several cases) to the Brazilian Ministry of Health.

#### Education and training

The clinical, diagnostic and research activity developed by the MGS-HCPA in the field of inborn errors of metabolism attracted the attention of many young fellows from Brazil and other Latin American countries, which are looking for training opportunities in the field. This lead to the creation of a comprehensive capacitation program in genetic metabolic diseases, developed under the umbrella of the WHO-CC activities. This program provides short (1 to 3 months) and long term (12 months) trainings, academic postgraduate courses (MSc or PhD programs with 2 to 4 years of duration), intensive short course and also scientific initiation opportunities. Table 1 summarizes the education and training opportunities provided by the reference center.

**Table 1:** Education and Training opportunities in genetic metabolic diseases offered by the IEM Reference Center

Type	Target audience	Hours per week	Duration	Options	Sponsors	Places per year
Intensive Training—Short term	Medical doctors, dietitians, biologists, biochemists, technicians	40 to 60	1 to 3 months	Metabolic Clinics, Biochemical genetics lab, Molecular genetics laboratory lab	Latin American Society of IEM, private companies, public agencies, trainee institutions	12
Intensive Training Long-term	Medical doctors with previous training in Pediatrics, Medical Genetics or Child Neurology	40 to 60	12 months	Metabolic clinics training and laboratory overview	Unrestricted educational grants from pharmaceutical companies	2
Advanced Course on Diagnosis and Treatment of Metabolic Diseases	Medical doctors, dietitians, biochemists, molecular biologists	60 h	One week	Lectures, case discussions and lab training	Unrestricted educational grants from pharmaceutical companies	25
MSc and PhD programs	Graduates from the biomedical courses, Health professionals	40 h	2 to 4 years	Credits from courses, dissertation or thesis	Fellowships granted by post-graduation agencies	8
Scientific Initiation	Undergraduate students	12 to 20	12 months (could be extended)	Clinics, laboratory, logistic, research	Research Agencies	6

#### Concluding remarks regarding the reference center activity on IEM

The “SIEM-Networks” combined strategy has enabled the diagnosis of thousands of cases of inborn errors if metabolism in Brazil since it was initially set up in 2001. With this strategy, sophisticated laboratories and skilled personnel located in a reference coordinating center were made available to medical services and physicians from all over the country, with the contribution of several associated centers in all Brazilian regions Also, a set of information tools (toll-free telephone, email, website, newsletter, family oriented booklets), educational actions (courses, symposia) and public activities were made available to increase the awareness about these diseases among health professionals, families and community. Patients with treatable conditions eventually get the treatment, in many instances by legal actions, as the Brazilian constitution guarantees access to healthcare for all citizens.

This model has been expanding more and more, serving as a template for new diagnostic networks in the metabolic (as the “MSUD Brazil Network”, for Maple Syrup Urine Disease) and non-metabolic (as the “RedeBRIM—Brazilian Network for Information and Reference in Microdeletion Syndromes); [7, 8] fields. Also, the SIEM example, in line with the older SIAT program, was used as a template for a new information program, the Hello Genetics (“Alô Genética”), aimed to provide information on genetic conditions to primary health care agents who work at community-based health centers [17]. Table 2 summarizes the different information and diagnostic services provided by MGS/HCPA.

**Table 2:** Information and diagnostic services provided by MGS/HCPA in the field of inborn errors of metabolism

Network	Main Goals	Target audience	Contact information	Started operation in	Average yearly rate of referrals	Average yearly rate of diagnosis
SIEM (Information Service on Metabolic Errors)	Provide information and guidance on diagnosis and urgent management of IEM	Health professionals	0800–510–2858 siem@ufrgs.br www.siem.ufrgs.br	2001	200	30
IEM Brazil Network	Provide information and diagnostic support in cases with suspected inborn error of intermediate metabolism	Health professionals, especially MDs	0800–645–2101 eim@ufrgs.br www.eim.ufrgs.br	2006	800	75
MPS Brazil Network	Provide information and diagnostic support in cases with suspected mucopolysaccharidoses	Health professionals, families and general public	0800–510–2030 mps@ufrgs.br www.mps.ufrgs.br	2004	700	65
DLD Brazil Network	Provide diagnostic support in cases of suspected lysosomal disease (except MPS or NPC)	Health professionals, especially MDs	0800–643–8011 dld@ufrgs.br www.dld.ufrgs.br	2012	700	20
NPC Brazil Network	Provide diagnostic support in cases of suspected Niemann-Pick disease type C	Health professionals, especially MDs	0800–643–8011 npc@ufrgs.br www.ufrgs.br/geneticahcpa/npc/	2010	250	15
DXB Network	Provide diagnostic support in cases of suspected Maple Syrup Urine Disease (MSUD)	Health professionals, especially MDs	0800–645–2101 faleconosco@redexaropedobordo.com.br http://redexaropedobordo.com.br/	2015	20	15

Because of the intensive diagnostic activity, a significant number of patients with very rare genetic metabolic diseases are being identified, which has enabled the development of several research projects. They include studies for the development of diagnostic methods more suitable for developing countries, for the better understanding of pathogenic mechanisms, for the development of new therapeutic alternatives and also clinical research protocols, most in association with other international centers, but some of them originally developed by Brazilian investigators, as the treatment of MPS I with encapsulated modified cell overexpressing alpha-iduronidase [1].

## Genetics, genomics and rare diseases

Brazilian genetics services consist of research associated with assistance to patients and families in a two-way road: patients contribute to new findings, while those findings help patients. In other words, clinical genetic services are to be offered as part of research and utilize funds acquired for such genetic investigations. The analysis of the description of the genetic services described in this article shows that, in spite of so many progresses, they maintain the same *modus operandi* implemented decades ago.

Public policies for rare diseases, both in the US and in Europe, originated from the pressure imposed by organizations of patients and families affected by specific diseases. In the US, in the 1970s, the “National Organization for Rare Disorders (NORD)”, a private organization, lobbied the government to create specific laws for the development of orphan drugs, which culminated in the “Orphan Drug Act” that established incentives for research on orphan drugs, including grants for clinical trials, market exclusivity and increased patent protection of the compounds [2]. Following the footsteps of this pioneer initiative, in the last two decades other countries such as Australia (1998), the European Union (2000) and Latin American countries like Colombia (2010) created a series of regulations which aimed the inclusion of rare diseases in health programs.

The vast majority of the population of Brazil depends exclusively on the public health system (SUS), and has still limited access to comprehensive genetic diagnosis and treatment, since there is not yet adequate coverage of genetic medical procedures through this system. In 2014, SUS designed “The National Policy for Rare Diseases”, establishing guidelines for offering treatment to individuals affected by rare diseases in the public unified health system. This is an appropriate policy for dealing with rare diseases which will finally bring medical genetics to the public health system. Unfortunately, up to now the policy has not left the printed page and it is still depending on the availability of funds to be effectively launched.

## Conclusions

Genetics and genomics have changed the medical practice in the last years, and more dramatic changes related to the new genetic technologies are expected to occur in the next years. With this view, it is highly desirable that medical genetics is formally incorporated into the health system in Brazil, and the move of the Ministry of Health with the national “Policy for the Integral Attention to Subjects with Rare Diseases” is surely in the right direction. While this does not occur, alternative approaches such as the ones developed for genetic metabolic diseases, are fulfilling the important role of providing services, promoting education and developing research in the area of inborn errors of metabolism in Brazil.
